# Enhancement of Apoptosis by Titanium Alloy Internal Fixations during Microwave Treatments for Fractures: An Animal Study

**DOI:** 10.1371/journal.pone.0132046

**Published:** 2015-07-01

**Authors:** Gang Wang, Yiming Xu, Lina Zhang, Dongmei Ye, Xianxuan Feng, Tengfei Fu, Yuehong Bai

**Affiliations:** 1 Department of Rehabilitation Medicine, Shanghai Jiao Tong University Affiliated Sixth People’s Hospital, Shanghai, China; 2 Department of Biostatistics, Shanghai Jiaotong University School of Medicine, Shanghai, China; Indian Institute of Technology, INDIA

## Abstract

**Objective:**

Microwaves are used in one method of physical therapy and can increase muscle tissue temperature which is useful for improving muscle, tendon and bone injuries. In the study, we sought to determine whether titanium alloy internal fixations influence apoptosis in tissues subjected to microwave treatments at 2,450 MHz and 40 W during the healing of fractures because this issue is not yet fully understood.

**Methods:**

In this study, titanium alloy internal fixations were used to treat 3.0-mm transverse osteotomies in the middle of New Zealand rabbits’ femurs. After the operation, 30-day microwave treatments were applied to the 3.0 mm transverse osteotomies 3 days after the operation. The changes in the temperatures of the muscle tissues in front of the implants or the 3.0 mm transverse osteotomies were measured during the microwave treatments. To characterize the effects of titanium alloy internal fixations on apoptosis in the muscles after microwave treatment, we performed TUNEL assays, fluorescent real-time (quantitative) PCR, western blotting analyses, reactive oxygen species (ROS) detection and transmission electron microscopy examinations.

**Results:**

The temperatures were markedly increased in the animals with the titanium alloy implants. Apoptosis in the muscle cells of the implanted group was significantly more extensive than that in the non-implanted control group at different time points. Transmission electron microscopy examinations of the skeletal muscles of the implanted groups revealed muscular mitochondrial swelling, vacuolization. ROS, Bax and Hsp70 were up-regulated, and Bcl-2 was down-regulated in the implanted group.

**Conclusion:**

Our results suggest that titanium alloy internal fixations caused greater muscular tissue cell apoptosis following 2,450 MHz, 40 W microwave treatments in this rabbit femur fracture models.

## Introduction

Microwave therapy is a common physical therapy method and can increase body temperature over 40°C, reduce pain [1, 2] and edema, stimulate the self-repair capacities of the tissues, and alter the physical properties of fibrous tissues [3]. Thus, physiotherapists apply microwave treatment for the curing and rehabilitation of muscle, tendon and bone injuries in the clinical. However, the use of microwave treatment has been contraindicated widely documented in the literature, and it could not be used if there is surgically implanted metal plate or screw in the presence of therapeutic areas. Microwave is a type of electromagnetic wave, and it can be refracted, reflected or transmitted at the boundary of the implants [4]. Further, the eddy current by electromagnetic stimulation can also cause Joule heating of the implants. Consequently, the temperatures of local tissues rapidly rise, and heat damages occurs [5,6]. Previous in vitro studies have shown that tissue ambustion can be caused by the temperature of a metal plate at frequencies near 900 MHz and 27 MHz [7]. However, in vitro studies of radio frequency (RF) electromagnetic fields showed that the metal implants caused little risk using 1800 MHz [8] and 2450 MHz [9,10] microwave radiation. Furthermore, shortwave diathermy, also a high frequency electrotherapy, was clinically applied by some doctors and therapist to the cure and rehabilitation in injuries bone with surgical implanted metal [11,12]. They found patients had no discomfort and pain. To evaluate the safety and efficacy of low-dose microwave on healing of fractures, we have found that 25 W microwave treatment resulted in significant improvements in the healing of fractures, and swelling myocytes were observed occasionally in the treatment field of the implanted group [13].

Cell death, particularly apoptosis, can occur after microwave exposure [14]. Two types of effects can be ascribed to microwaves, i.e. thermal and non-thermal. The thermal effect is due to the transformation of electromagnetic energy into heat [15–18]. Hyperthermia can induce apoptosis that is mediated by the mitochondria. The regulation of mitochondrion-mediated apoptosis is based on the intracellular dominance of various proteins that induce or inhibit apoptosis, such as Bax, Bcl and several key enzymes. Titanium alloy internal fixations can reflect microwaves to increase the temperatures of tissues within the field of the microwave treatment, and the safety limit of microwave treatment is unknown. Besides, the influence following 2,450 MHz, 40 W microwave treatment that could be used in titanium alloy-fixed bone fracture has not been identified. The research hypothesis of this study was that 40 W microwave treatments would cause more damage to muscle tissues near titanium alloy internal fixations in the healing of fractures. To address this question, our study performed TUNEL assays, reactive oxygen species (ROS) detection, fluorescent real-time (quantitative) PCR, western blotting analyses and transmission electron microscopy examinations that were related with muscle cell apoptosis.

## Materials and Methods

### Ethics statement

This study was carried out in strict accordance with the recommendations in the Guide for the Care and Use of Laboratory Animals of National Laboratory Animals Regulations. The protocol was approved by the Committee on the Ethics of Animal Experiments of Shanghai Jiao Tong University Affiliated Sixth People’s Hospital (Permit Number: SYXK(HU) 2011–0128). All surgery was performed under sodium pentobarbital anesthesia, and all efforts were made to minimize suffering.

### Animals

Fifty-four male healthy New Zealand adult white rabbits were used in this experiment, and they weighed 2.0 and 3.2 kilograms (average: 2.5 kilograms). The rabbits were fed in the Animal Laboratory Center of Shanghai Jiao Tong University Affiliated Sixth People’s Hospital, and the Animal Laboratory Center had a light, humidity, and temperature controlled environment.

**Fig 1:**
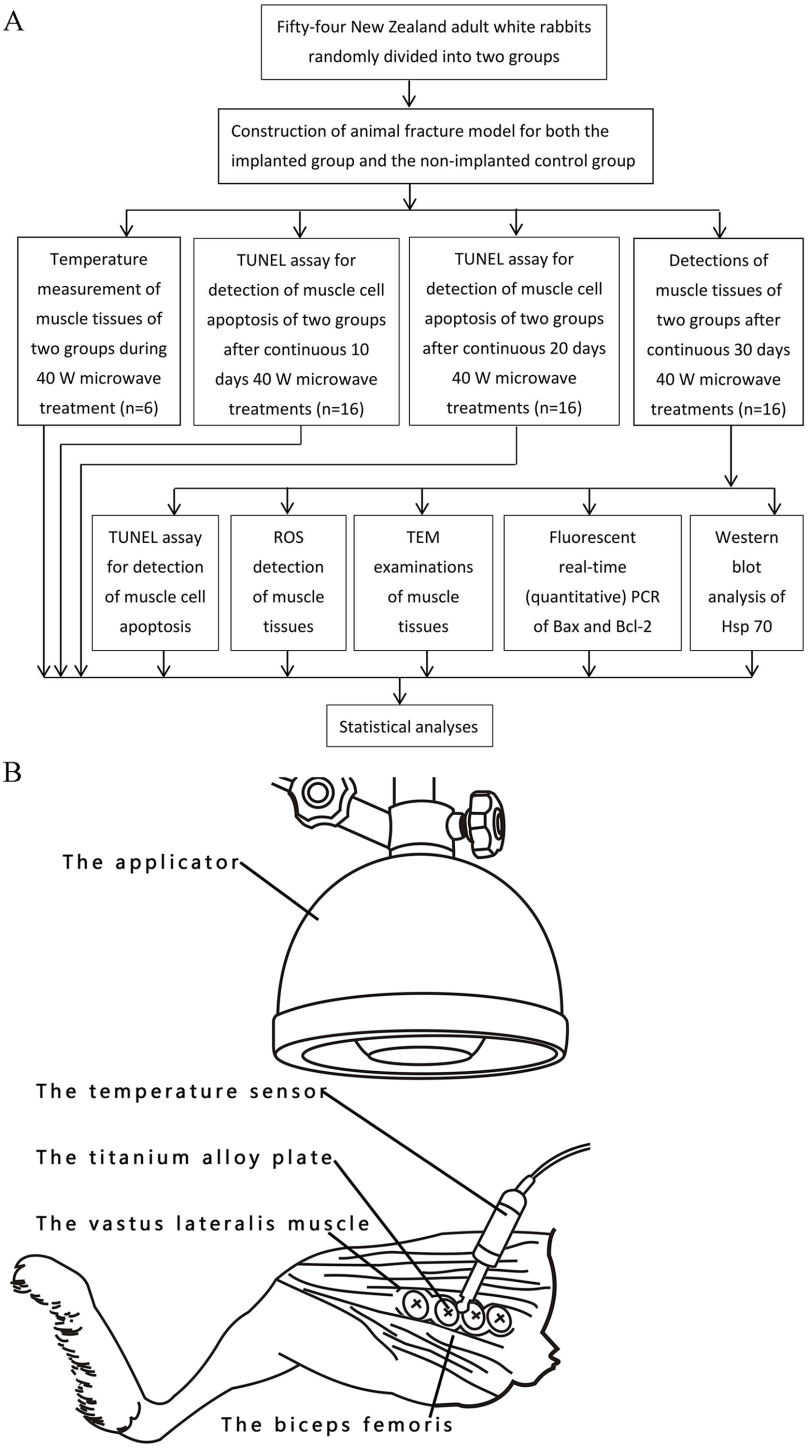
The flow with the whole protocol (A) and temperature measurement in the implanted group (B).

The rabbits were randomly divided into two groups a week of adaptation later. The non-implanted control group included 27 control rabbits without titanium alloy implants on 3.0 mm transverse osteotomies, and the implanted group also included 27 rabbits that were surgically implanted using titanium alloy implants on their 3.0 mm transverse osteotomies. The two groups received microwave treatments following the surgeries. There is a flow with the whole protocol in the Fig 1A.

### Construction of animal fracture model

The animals of two groups were anesthetized using intravenous injections of sodium pentobarbital (30 mg/kg). Lateral longitudinal incision of the right hind limb skin was used to expose the femur. In this study, 3.0 mm transverse osteotomies were used as fractures. Internal fixation system (LCP, Synthes Company, USA) included titanium plate (4.58 ± 0.23 X0.41 ± 0.08 cm) and screws, and the system was used in the right femur of each rabbit of the implanted group to treat the 3.0 mm transverse osteotomies. As a control, each rabbit of the non-implanted control group was also subjected to the same procedure, but not used the titanium alloy implants to treat the 3.0 mm transverse osteotomies. The 3.0 mm transverse osteotomies produced no serious damage to the femur, and the fractures of the control groups were not fixed. To prevent infection and reduce postoperative pain, each rabbit of two groups was intramuscularly injected using penicillin (800,000 units). Following the surgeries, surgical dressings were applied to the incisions once a day for 3 days.

### Treatment of microwave

Microwave therapy was applied to both groups three days after the operations. The right upper thigh was the treatment regimen. The applicator (RM-170A, ITO Company, Japan) used in this study was attached to a microwave generator (PM-800, ITO Company, Japan). The generator had a power output from 0 to 200 W at frequencies 2,450 MHz. In previous experiments, Ye D et al. [13] found that 25 W microwave treatment did not induce irreversible damage to the muscle around titanium alloy internal fixations and resulted in significant improvements in the healing of fractures. 40 W is a usual therapeutic dose of microwave therapy for deep tissues in clinical [4]. However, the safe of 40 W microwave treatment that can be used in titanium alloy-fixed bone fracture has not been identified. To address this question, each rabbit received continuous-wave 40 W microwave treatment for 20 min every day in this study. The distance between the lesion and the non-contact applicator of treatment was 10 centimeters. The non-contact applicator was vertical to the skin. The rabbits received the microwave treatments at the same time of the day to exclude the impact of the daily temperature changes of the rabbits and other factors.

### Measurement of the temperatures

Three rabbits from two groups were used to measure temperatures. In this study, a couple thermometer (FHC, ME-04008, Bowdoinham, USA) was used for the measurements of temperatures during the microwave treatments. An intravenous injection of sodium pentobarbital (30 mg/kg) was used to anesthetize the rabbits of two groups. After anesthesia, an 8 centimeter-long thermal probe was placed into the muscles at 5 mm above the 3.0 mm transverse osteotomies or the middle of the titanium alloy implants to measure the temperatures. We separated the biceps femoris and the vastus lateralis muscles by blunt dissection to place the thermal probe between the muscles, and the recording side of the probe was placed toward the biceps femoris (Fig 1B). In the same method, the rabbits of the non-implanted control group were measured for the temperatures. In the 20 minutes treatment process of the 40 W microwave treatments, the temperatures of the muscles were recorded every minute. Laboratory temperature was maintained at 24°C in the temperature measures.

After the temperature measures, the influence of the titanium alloy implants in the continuous 30-day microwave treatments to apoptosis of muscles was studied. On the day following the last microwave treatments of continuous 30 days, the skeleton muscle tissues in front of the titanium alloy implants or the 3.0 mm transverse osteotomies were examined with TUNEL assays, transmission electron microscopy examinations, ROS detection, fluorescent real-time (quantitative) PCR and western-blot analyses for apoptosis. To explore the trend in the development of the muscle cell apoptosis, TUNEL assays were also performed after the 10-day and 20-day microwave treatments.

### The TUNEL assay for detection of apoptosis

Apoptosis in the muscles was examined after the 10-day, 20-day and 30-day microwave treatments. Eight rabbits from each group at 10 days, 20 days and 30 days following the microwave therapy were sacrificed under anesthesia with intravenous injections of sodium pentobarbital (30 mg/kg). After euthanasia via air embolism under anesthesia with an intravenous injection of sodium pentobarbital (30 mg/kg), the muscle tissues in front of the titanium alloy internal fixations or the 3.0 mm transverse osteotomies were obtained and fixed in 10%formalin overnight at room temperature for TUNEL staining. A commercial kit (In Situ Cell Death Detection Kit, POD, Roche Molecular Biochemicals, Mannheim, Germany) that links digoxigenin- nucleotides to DNA by TdT was used. Five micrometer thick sections were deparaffined with xylene, rehydrated in a descending series of ethanol, incubated with proteinase K, immersed in 3% aqueous hydrogen peroxide, and then pretreated with equilibration buffer. DNA was labeled at the 3′-end by incubating the sections with a mixture of FITC deoxynucleotide triphosphate, unlabeled deoxynucleotide triphosphate, and TdT enzyme at 37°C for 1 h. The slides were washed with phosphate buffered saline (PBS) and incubated with anti-FITC antibody conjugated to peroxidase at room temperature for 30 min. The slides were washed again in PBS, incubated with 3, 3-diaminobenzidine peroxidase substrate, counterstained with hematoxylin, mounted, and sealed. Using an ocular grid at 40× magnification of light microscopy, the percentages of TUNEL-positive muscle cell nuclei over scoring 100 scored cells was calculated as the apoptotic indices of the muscle tissues.

### Transmission electron microscopy examinations

After 30-day microwave treatments, the samples of the muscular tissues in front of the implants or the fractures without implants were fixed for 16 h at 4°C in 2.5% glutaraldehyde, and then post-fixed for 2 h at 4°C in 2% OsO4. After fixations, they were dehydrated in an ascending ethanol series, and then passed through propylene oxide, finally embedded in resin. Ultrathin sections (90 nm thick) on mesh grids were stained with uranyl acetate and lead acetate and examined with an electron microscope. For quantitative analyses of the mitochondrial damage in the muscles in two implanted groups, we calculated the percentage of damaged mitochondrial area in each sample using the KS400 image analysis system (Zeiss).

### Detection of reactive oxygen species

After 30-day microwave treatments, frozen sections of muscle tissues were cut to a thickness of 10 μm at optimized cutting temperature. Mounted slides were incubated with Dihydroethidium (DHE) (100 μmol/L; Beyotime Institute of Biotechnology, Jiangsu, China) for 30 min at 37°C in the dark, after which excessive reagent was rinsed off. Tissue sections were then visualized with a Zeiss 710 confocal microscope, and fluorescence was detected with a 590-nm long-pass filter.

### Fluorescent real-time (quantitative) PCR of the genes

After 30-day microwave treatments, each specimen of RNA was extracted from the muscular tissues around the implants or the fractures without implants using Trizol reagent (Gibco). Then, using a First Strand cDNA Synthesis Kit (TaKaRa), they were reversed transcribed. For the real-time PCR analyses, an ABI 7300 Detector System (Applied Biosystems, USA) was applied. Using SYBR Premix Ex TaqTM II (TaKaRa), fluorescence signals were produced via the 5'–3' endonuclease activity of Taq during each PCR cycle. The PCR was performed using the following conditions: initial denaturation at 95°C for 10s, followed by 40 cycles of denaturation at 95°C for 5s, and annealing, and extension at 60°C for 31 s. All of the experiments were performed five times. The following primers were applied in this study: GAPDH (forward, 5'- GAA GGT CGG AGT CAA CGG AT-3'; reverse, 5'-CCT GGA AGA TGG TGA TGG G-3'); Bax (forward, 5'-CAG GAT GCG TCC ACC AAG AA-3'; reverse, 5'-CCA GTT GAA GTT GCC GTC AGA-3'); and BCL-2 (forward, 5'-CAT TGG GAA GTT TCA AAT CAG C-3'; reverse, 5'-CTT GGC ATG AGA TGC AGG AA-3'). The reactions were performed in a 20μl reaction volume containing 2μl of cDNA, 9 μl of SYBR Premix Ex TaqTM II, and 1 μl each of the forward and reverse primers. Under the method of the double standard curve, the relative quantification was carried out on an ABI 7300 Detector System with the ABI 7300 SDS Software (Version 1.2). The quantitative results of two groups are expressed as means ± the standard deviations.

### Western blot analysis

After 30-day microwave treatments, total protein was extracted and analyzed with a bicinchoninic acid protein concentration assay kit (Beyotime, China). Protein samples (30μg) were boiled with 5×sample buffer, electrophoresed on polyacrylamide gels, and transferred to nitrocellulose membranes. The membranes were washed and blocked and incubated with antibodies to detect HSP70 (1: 1000; Abcam, Cambridge UK) for 12 h at 4°C. HRPlinked secondary antibody (1: 5000; Abcam, Cambridge, UK) was added for 40min at room temperature. The membranes were washed and visualized by autoradiography after development with an ECL Plus Kit (Millipore MIT, USA). β-actin was used as internal control. Densitometry was performed with gel documentation equipment (Gel Doc 2000, Quantity One, Bio-Rad, Hercules, CA).

### Statistics

The statistical analyses of the temperatures were performed a repeated measures analyses of variance with SAS 9.1 for Windows, and the others analyses were carried out using SPSS 17.0 for Windows. With regard to the SPSS analyses, all of the results are presented as the mean value ± the standard deviation (SD). The differences between groups were tested using t-tests. For all analyses, the *P* values were two-tailed, and *P* <0.05 was considered statistically significant.

## Results

### Temperature changes

**Fig 2:**
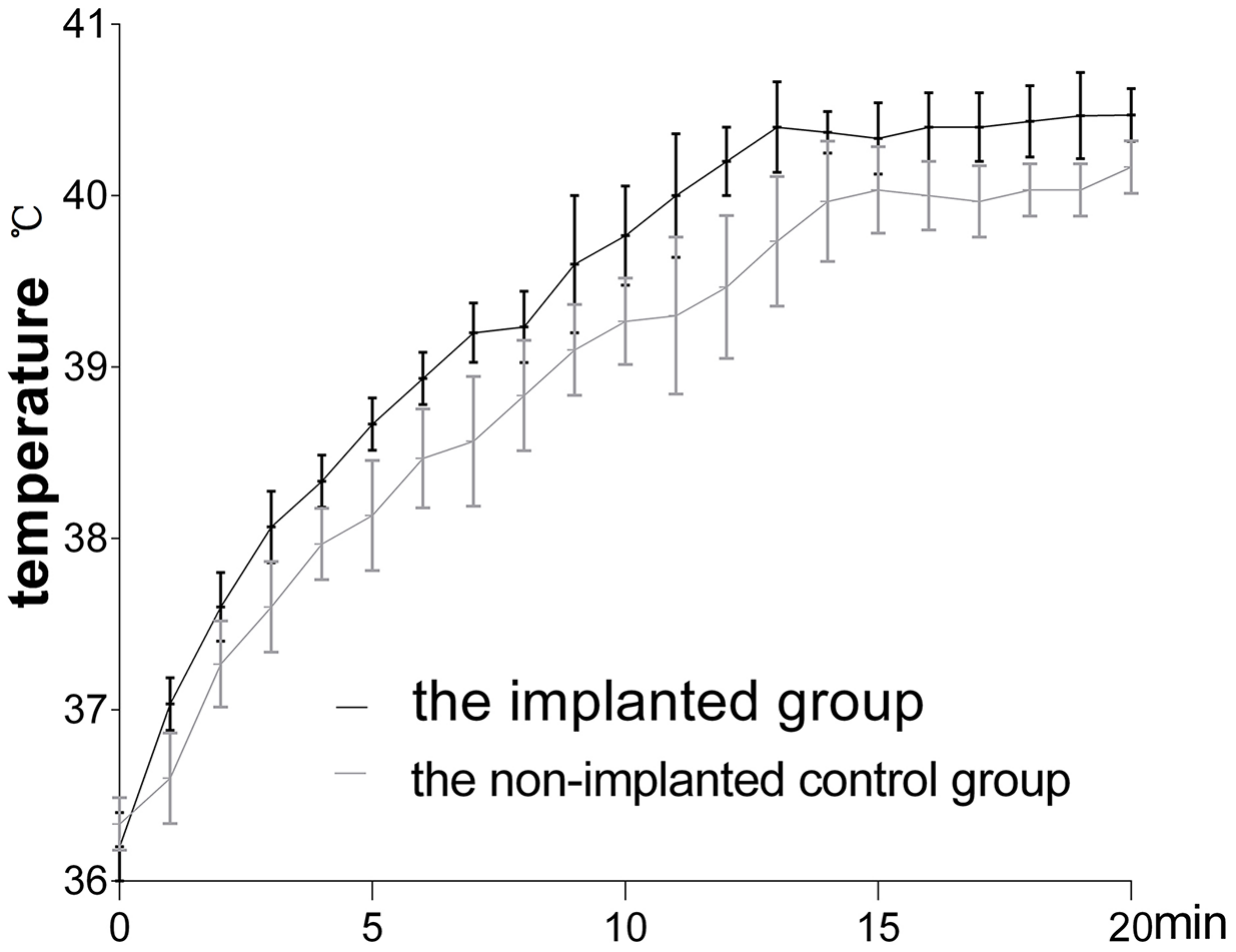
The temperature changes of muscle tissues during microwave treatment. The temperatures of the implanted group (the black line) in muscle tissues were higher than those of the non-implanted control group (the gray line), and the difference between two groups was statistically significant.

During the microwave irradiation, we measured the temperatures in the muscle tissues of two groups. The changes of the temperatures are show in Fig 2. Using a repeated measures analyses of variance with SAS 9.1, we identified a significant difference between the implanted group and the non-implanted control group at 20 minutes (*P* = 0.0373).

### Skeleton muscle cell apoptosis

**Fig 3:**
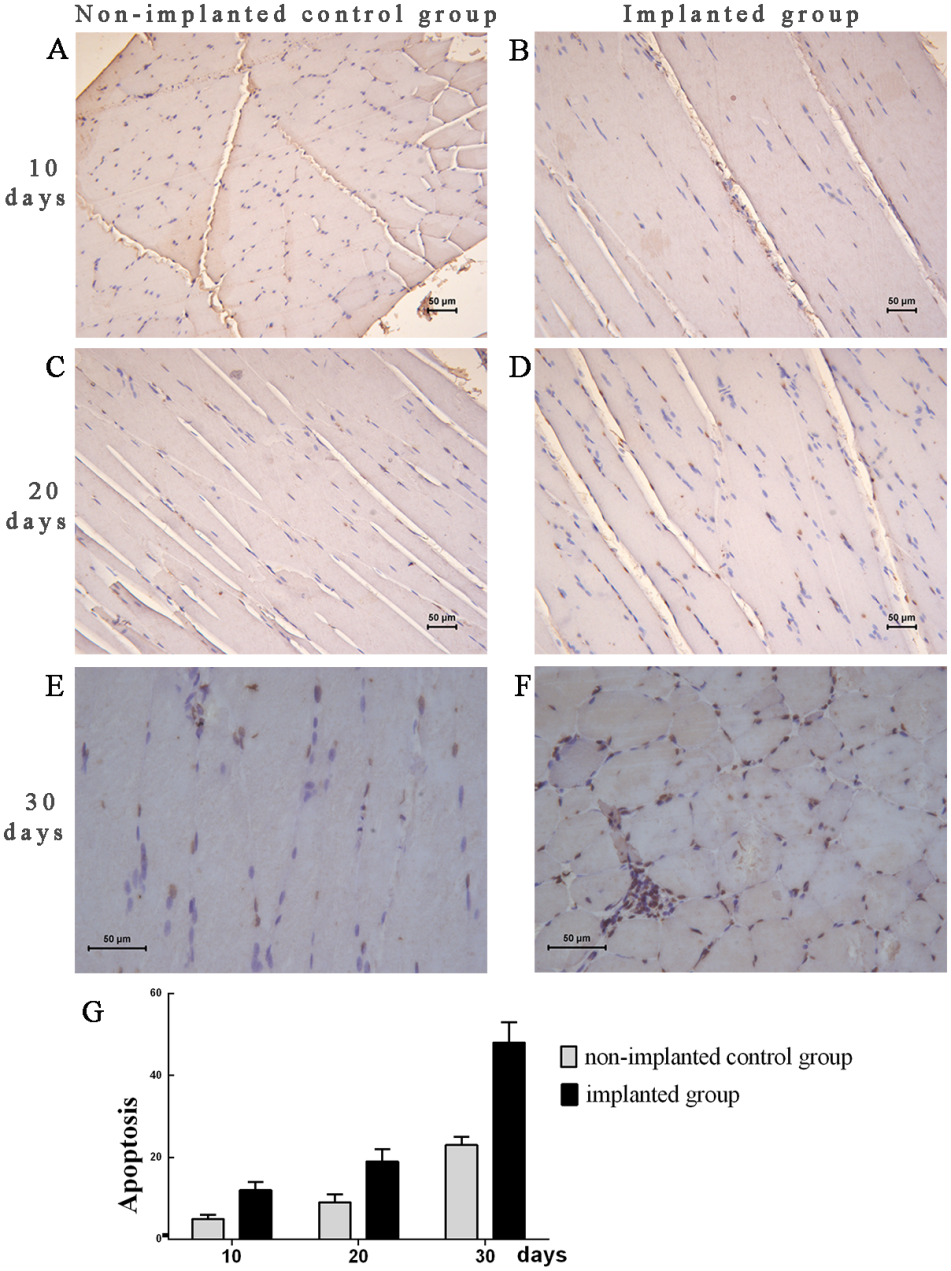
Skeleton muscle cell apoptosis. The nuclei of apoptotic cells are dark brown in both groups. There were fewer apoptotic cells in the non-implanted control group (B, D and F) than in the implanted group (A, C and E) at 10, 20 and 30 days. (G) Compared to the non-implanted control group, the apoptotic index of the implanted group was significantly increased (*P* < 0.01). The data were collected from the specimens of both groups at different time points. Scale bars: 50 μm (A, B).

The titanium alloy internal fixations significantly increased the numbers of apoptotic cells when observed 10 days, 20 days and 30 days after the treatment (Fig 3B, 3D and 3F). Without the titanium alloy implants, the skeletal muscle cells of the non-implanted group underwent apoptosis as revealed by TUNEL assay (Fig 3A, 3C and 3E). Fig 3G shows that the number of apoptotic cells in the implanted group induced by microwave treatment was significantly greater than that the non-implanted group at three time points (*P* < 0.01), and gradually increased with the extension of time.

### Transmission electron microscopy examinations

**Fig 4:**
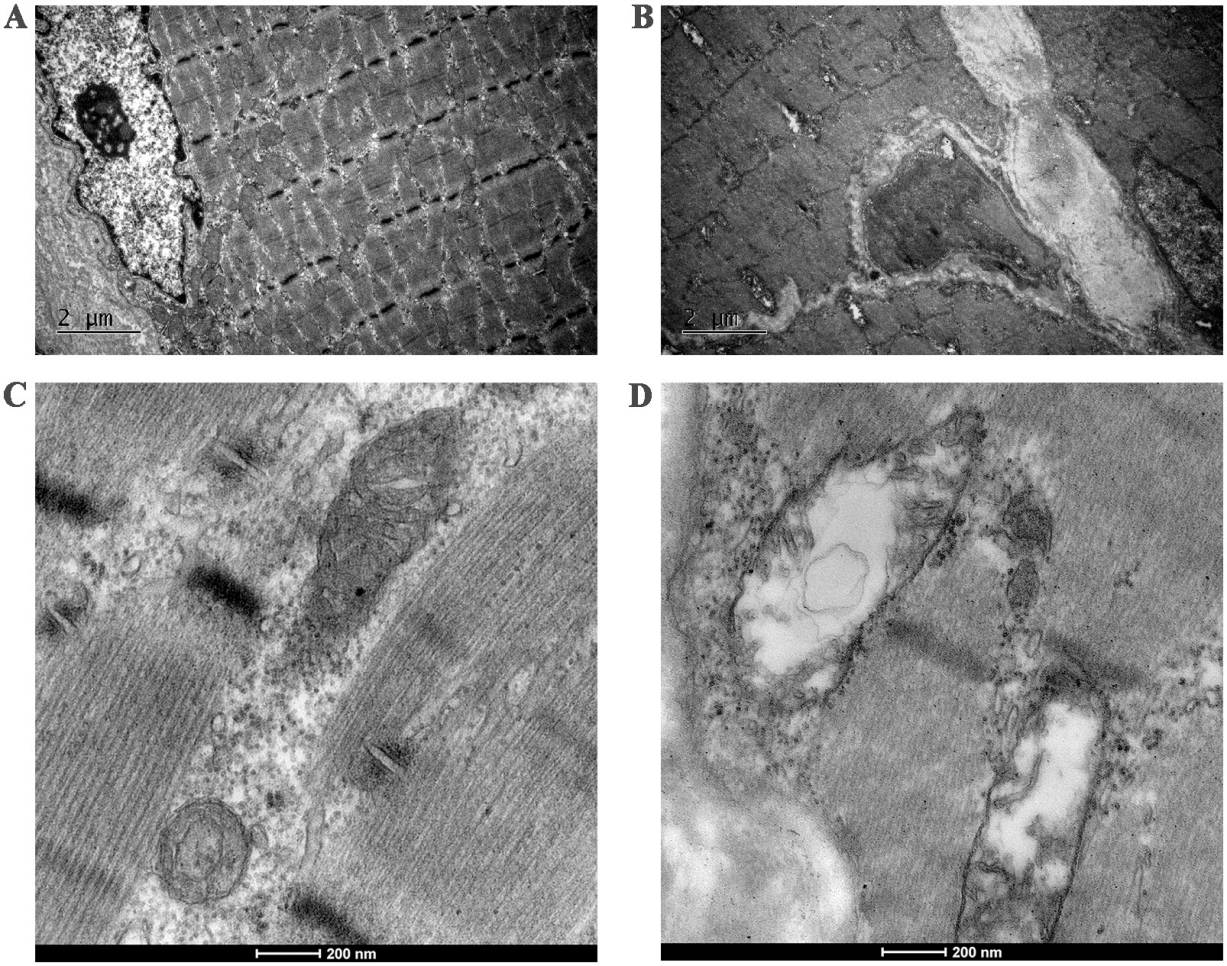
Transmission electron microscopy photographs of thigh muscles. B and D show that the mitochondrial swelling, partial deletion of mitochondrial crest and vacuolar changes in the implanted group. (B) Additionally, the myofilaments and myocommata are unclear. (A and C) There were no abnormal morphological changes in the non-implanted control group. Scale bars: 2 μm (A, B), 200 nm (C and D).

Transmission electron microscopy examinations of the muscle tissues in front of the implants or the 3.0 mm transverse osteotomies from two groups were conducted after the 30-day microwave treatments. The muscles of the implanted group at treated with 40 W (Fig 4B and 4D) exhibited mitochondrial swelling, partial deletion of mitochondrial crest and vacuolization (mitochondrial damage). The myofilaments and myocommata were also unclear (Fig 4B). There were no abnormal morphological changes observed in the non-implanted control group (Fig 4A and 4C). The scale bars indicate: 2 μm (Fig 4A and 4B), 200 nm (Fig 4C and 4D). There was a significant difference in the mitochondrial damage to the muscles between two groups, and the percentage of mitochondrial damage in the implanted group was 31%±5%.

### Effects of microwave treatments on muscle ROS production

**Fig 5:**
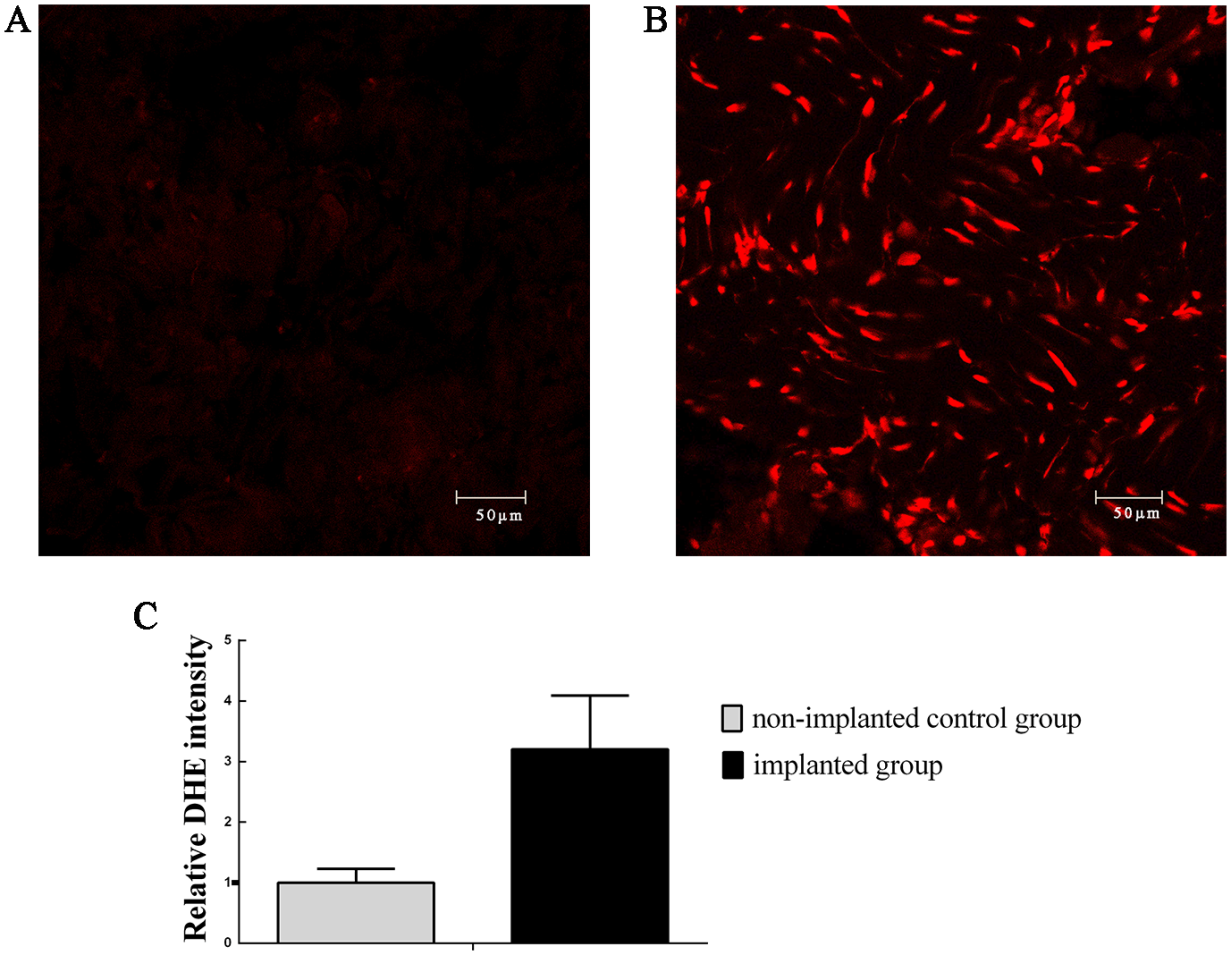
ROS detection of thigh muscles. ROS (indicated by red fluorescence) was dramatically higher in the muscle tissue of the implanted group (B) than in that of the non-implanted control group (A). (C) The intensity of red fluorescence was assessed by Image-Pro Plus software (Media Cybernetics Inc., Bethesda, MD, USA), and the implanted group showed significantly increased ROS levels in muscle tissue (*P* < 0.05). Scale bars: 50 μm (A, B).

Reactive oxygen species production was assessed in situ by DHE staining. As shown in Fig 5, ROS (indicated by red fluorescence) were dramatically higher in the muscle tissue of the implanted group (Fig 5B) than in that of the non-implanted control group (Fig 5A) after 30-day microwave treatments. The implanted group showed significantly increased ROS levels in muscle tissue (Fig 5C) (*P* < 0.05 vs. the non-implanted control group).

### Transcriptional expressions of two genes

**Fig 6:**
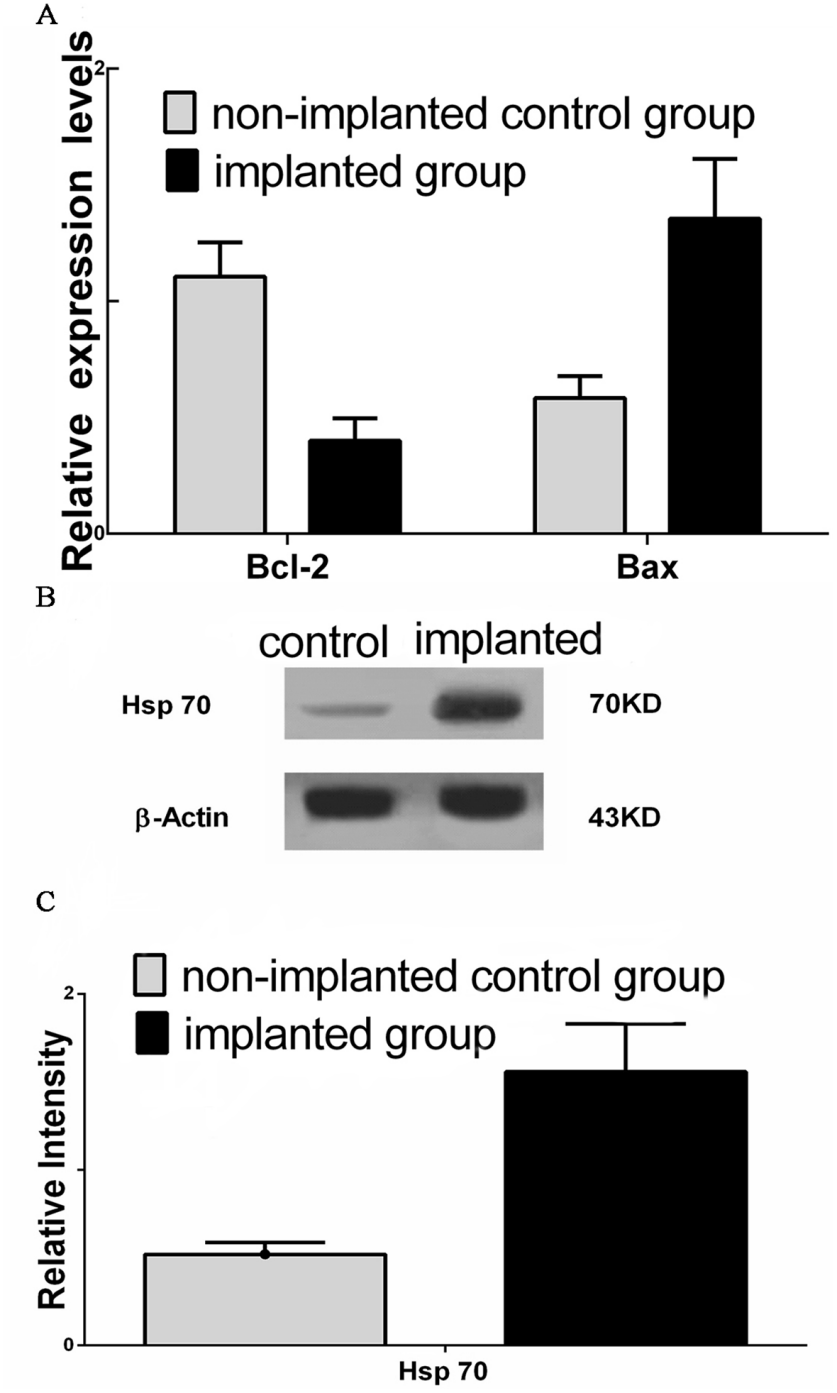
Relative expression levels of the Bax and Bcl-2 mRNAs and the western blot analysis of Hsp70 (mean±SD). (A) The expression level of Bcl-2 reduced significantly in the implanted group compared with in the non-implanted control group (*P* < 0.01). Additionally, the expression levels of Bax were remarkably increased in the implanted group compared to the in the non-implanted control group (*P* < 0.01). (B,C) Hsp70 proteins were more highly expressed in the implanted group than in the non-implanted control group (*P* < 0.01). The data are presented as the means ± the SDs (n = 8). The vertical bars represent the SD.

The level of anti-apoptotic Bcl-2 transcripts was much lower in the implanted control group tissues than in the non-implanted control group (Fig 6A). In contrast, there were significantly more Bax transcripts in the implanted control group tissues than in the non-implanted control group. The transcriptional expressions of Bcl-2 and Bax in the implanted control group were significantly different from those of the non-implanted control group (*P* < 0.01).

### Expression of Hsp70

The expressions of heat shock protein 70 in the two groups following the 30-day microwave treatments are shown in Fig 6B and 6C. The statistical analyses revealed that Hsp70 expression was higher in the implanted group than in the non-implanted control group (*P* < 0.01).

## Discussion

In general, the metallic implant in potential treatment regions is a contraindication for radio frequency due to the occurrence of intense heating. Therefore, the purpose of the present study was to evaluate the safety of titanium alloy internal fixations subjected to 40 W microwave treatments for fractures.

In this study, repeated measures analysis of variance with SAS 9.1 revealed a significant difference in the temperatures between two groups at 20 minutes; however, this treatment did not significantly elevate the temperatures of the muscle tissues at any time point. Due to the cumulative effect of various temperatures, the temperatures were different between the two groups. The increase of temperature was one reason for apoptosis of muscle cells. There might be other ones, such as the reactive oxygen species or other non-thermal effects of microwave. Several studies demonstrated that microwave could cause cells apoptosis and the temperature was under 41°C [19–21]. The mechanism of muscle cell apoptosis in this study was complicated, and not exactly the same as that of tumor cell apoptosis in hyperthermia condition [22]. Two types of effects can be ascribed to microwaves, i.e. thermal and non-thermal. The thermal effect is the major role in treatment and due to the transformation of electromagnetic energy into heat [4,23]. Titanium alloy internal fixation can increase these two effects of microwave therapy. The implant may reflect microwave and a current is induced on the implant surface. The induced current further produces a secondary electromagnetic field and so the implant acts as weak radiating antenna in tissues [8]. Cells that are undergone to hyperthermia can occur apoptosis or necrosis, and it relates with temperature of the tissue and the time interval of the treatment (CEM_43_ = Δt * R ^43-T^). As described in detail previously [24], the critical temperature of 43°C is generally considered to the threshold. If the temperature is beyond 43°C, more cell death occurs. Histological examinations of the skeletal muscles in pigs have showed that the minor damage can occur at 30 CEM_43_ [25]. A thermal dose scale of 240 CEM_43_ has been established for the leg muscles of rabbits and pigs and this dose is an irreversible lethal dose to the tissue cells [26–28]. In the implanted group, the results revealed that the temperature increases in the muscles in front of the titanium alloy implants were less than 41°C. The thermal dose (CEM_43_) of the implanted group was 0.31 CEM_43_. When applied to the subjects with titanium alloy implants in this study, this dose of microwave treatments caused apoptosis in the muscles around the implants. In previous experiments, we found that 25 W microwave treatment did not cause damage to the bone tissues and cells around titanium alloy internal fixations between the implanted and the non-implanted control group [13]. TUNEL assays of bone tissues also showed that 40 W microwave treatment also did not cause obvious damage to the bone cells in both the implanted group and the non-implanted control group (S1 Fig).

The TUNEL (terminal deoxynucleotidyl transferase-mediated dUTP nick-end labeling) technique has been described as a sensitive method for the detection of apoptotic nuclei in tissues and the preferential staining of apoptotic strand breaks. In our study, the numbers of apoptotic cells in the implanted group were influenced by titanium alloy implants and were significantly greater in the implanted group than in the non-implanted group as assessed with TUNEL assay and transmission electron microscopy of the implanted group revealed mitochondrial swelling and vacuolization (i.e., mitochondrial damage). Besides, Reactive oxygen species (ROS) were dramatically higher in the muscle tissues of the implanted group than in those of the non-implanted control group in this study. ROS get involved in many normal cellular functions such as proliferation, signaling pathways, and apoptosis [29]. Cells and tissues are equipped with a extensive range of antioxidant defense systems that balance the output of ROS under normal conditions. Heat stress was also thought to decrease superoxide dismutase 1 (SOD-1) mRNA levels, cytoplasmic SOD protein and enzyme activity, resulting in the increase of ROS generation. Furthermore, heat stress causes an over-production of transition metal ions, which can make electron donations to oxygen, forming superoxide anions [30,31]. Mitochondria as major ROS generators are usually the aims of high ROS exposure with destructive results, such as causing severe damage to cellular lipids, proteins and DNA [29]. ROS overproduction can result in the opening of the mitochondrial permeability transition pore [32] and the deprivation of Mitochondrial membrane potential [33]. In our study, the mitochondria of the muscles near the implants were swollen, and those of the control group were not swollen. The unclear myofilaments and myocommata resulted from accelerated blood circulation and increased tissue fluid exudation into the muscle cells due to the elevated temperature. Previous studies have found that microwave radition cause changes in the mitochondria [34,35]. Mitochondria play an integral role in apoptotic cell death. Mitochondrial swelling is the most important indicator of the mitochondrial permeability transition pore opening. The opening of the mitochondrial permeability transition pore or mitochondrial swelling can make the mitochondrial outer membrane channel rupture, leading to the release of cytochrome c, and eventually leading to necrosis or apoptotic cell death [36].

Hyperthermia induced by microwave treatment can play a role in treatment and can enhance apoptosis. It is widely known that apoptosis is primarily regulated by death receptor pathways and mitochondria. Hyperthermia-induced apoptosis is thought to be mediated by the intrinsic mitochondrial pathway rather than the extrinsic death receptor pathway. The early stage of apoptosis involves many death-inducing signals, such the ligands of the death receptors, imbalances in calcium regulation, reactive oxygen and nitrogen species, and alterations in the composition and abundance of B-cell lymphoma (Bcl)-2 family proteins, such as Bax, Bad, Bcl-2, and Bcl-xl [37]. Cell apoptosis and the apoptosis that is related to the expression of genes and proteins are closely related. The Bcl-2 gene family is generally considered as the important regulator of apoptosis. As described in detail previously [38], the inherent susceptibility of the reaction to the apoptotic signals for cells is determined by the changes in the expression of the anti-apoptotic proteins and pro-apoptotic proteins of the Bcl-2 gene family. The anti-apoptotic Bcl-2 proteins and the pro-apoptotic Bax are the significant proteins in the Bcl-2 family, and the rate of Bcl-2/Bax is crucial to apoptosis cell death [38]. Bcl-2 from the mitochondria inhibits the release of cytochrome c, and Bax inactivates the caspase cascade and thus prevents stress-induced apoptosis [39,40]. If this balance is disrupted by stress, cytochrome c is released from the mitochondria, the activity of Bax increases, and caspases are cleaved and activated to induce apoptosis [41]. In our experiment, the transcripts of anti-apoptotic Bcl-2 were reduced to a much lesser extent in the tissues of the implanted control group that were subjected to microwave treatment. Additionally, there were many more Bax transcripts in the implanted control group tissues that were subjected to microwave treatment. Thus, the difference in the Bcl-2/Bax ratio of the mitochondria between two groups might have been the reason for the muscular cell apoptosis.

Hsp70 is induced by stimuli such as hypoxia, cellular damage, hyperthermia and oxidative stress [42]. The biochemical alterations induced by HSPs and anti-apoptotic factors in response to hyperthermia have not been fully defined. Although it is well known that hyperthermia is damaging and induces apoptosis, the defences against hyperthermia are not fully understood. Once cells are exposed to several stressors, including hyperthermia, heat shock protein genes, such as Hsp70 are expressed. Thus, heat damage was related to the expression of Hsp70. Hsp70 functions as a chaperone that inhibits apoptosis [43] by blocking release of Bax from mitochondria, inhibiting the formation of apoptosome complexes, activating of Bid and inducing the migration of AIF from the mitochondria to the nucleus [44]. In our study, the expression of Hsp70 was significantly increased in the implanted group compared to the non-implanted control group, which is consistent with the changes in temperatures. Thus, the titanium alloy internal fixation caused much more heat damage to the muscle during the 40 W microwave treatments.

This study had some limitations. First, the feelings during the microwave treatments such as pain could not be obtained from the animals in the experiment. Second, this study did not discuss the long-term effects (3 months, 6months or 1 year) of titanium alloy implants for fractures after 30-day microwave treatments. Third, this study discussed what happened in the tissues around the fracture with titanium alloy internal fixations within the 30 days, but it did not find out when the earliest tissue cell apoptosis appeared and what would be happened if the microwave therapy lasted for short time. There has no article that discussed the type of heat damage caused by titanium alloy internal fixations during 2,450 MHz, 40 W microwave treatments, and this is only a theoretical rabbit femur fracture model experiment. So the safe and effective parameter of microwave that can be used in the clinical titanium alloy-fixed bone fracture needs much further studies.

In conclusion, our *in vivo* study proved that titanium alloy internal fixation dramatically increased the temperature in the muscle tissues during the continuous-wave microwave treatment at 2,450 MHz, 40 W and 20 minutes per day. The titanium alloy implants induced more apoptosis of muscle tissue cells, and this apoptosis was related with the mitochondria. This study can help to choose the appropriate microwave treatment to heal fractures with titanium alloy internal fixations.

## Supporting Information

S1 Fig TUNEL assays of the treated femurs adjacent to the implants after 30-day microwave treatment. The nuclei of apoptotic cells are dark brown in both groups. Apoptotic bone cells were rarely observed in both the implanted group (B) and the non-implanted control group (A). Scale bars: 50 μm.
(TIF)
