# Supplementary figures and images for: Enhancement of Apoptosis by Titanium Alloy Internal Fixations during Microwave Treatments for Fractures: An Animal Study
Source: PLoS One. 2015 Jul 1;10(7):e0132046. doi: 10.1371/journal.pone.0132046 (PMC4488932; doi:10.1371/journal.pone.0132046)

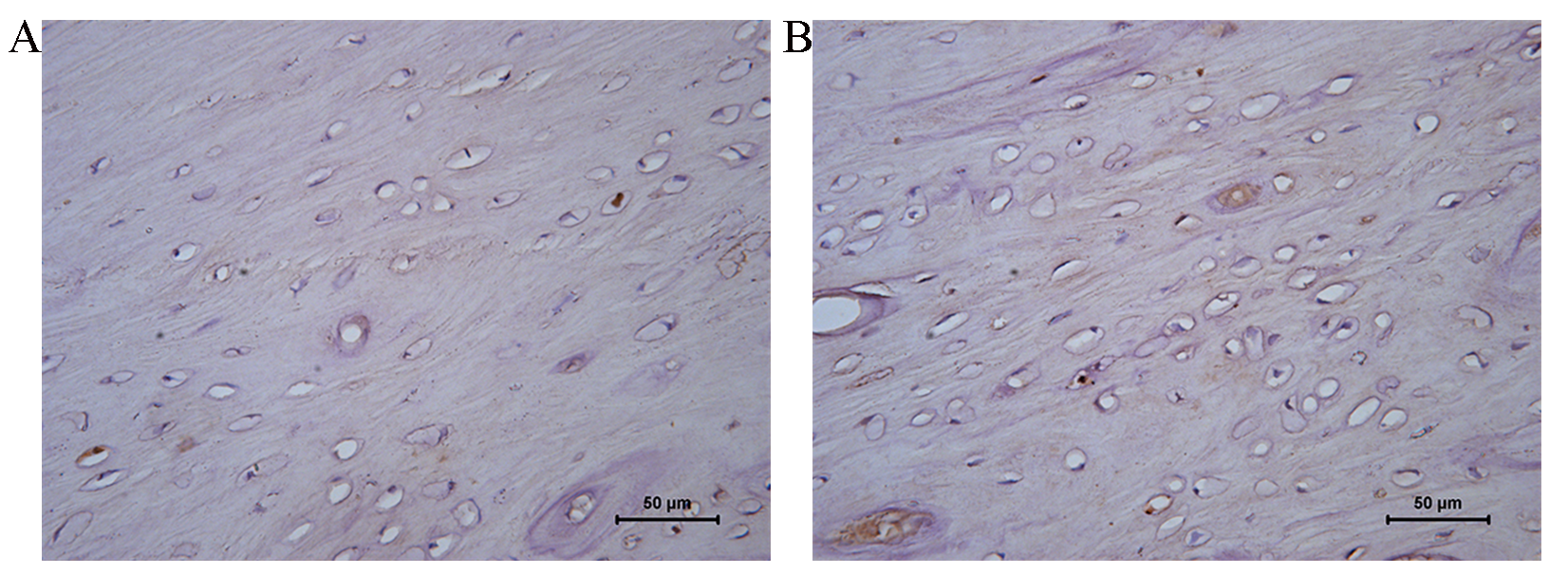

Supplement: S1 Fig — The nuclei of apoptotic cells are dark brown in both groups. Apoptotic bone cells were rarely observed in both the implanted group (B) and the non-implanted control group (A). Scale bars: 50 μm. (TIF) [file pone.0132046.s001.tif]
